# Mismatch Repair Proteins and CD45RO-Positive Lymphocytes in Malignant and Benign Salivary Gland Tumors: A Favorable Association with Disease Clinical Parameters

**DOI:** 10.1055/s-0045-1806721

**Published:** 2026-03-17

**Authors:** Hamid Ghaderi, Yousef Mohammadi, Simin Ahmadvand, Akbar Safaei, Sajjad Gerdabi, Fatemeh Asadian, Bijan Khademi, Mohammad Reza Haghshenas, Abbas Ghaderi

**Affiliations:** 1 Violet Vines Marshman Centre for Rural Health Research, La Trobe Rural Health School, La Trobe University Bendigo, Bendigo, Victoria, Australia; 2 School of Medicine, Shiraz Institute for Cancer Research, Shiraz University of Medical Sciences, Shiraz, Iran; 3 Department of Biochemistry, Microbiology, and Immunology, Faculty of Medicine, University of Ottawa, Ottawa, ON, Canada; 4 Department of Pathology, School of Medicine, Shiraz University of Medical Sciences, Shiraz, Iran; 5 Department of Medical Laboratory Sciences, School of Paramedical Sciences, Shiraz University of Medical Sciences, Shiraz, Iran; 6 Department of Otolaryngology, Otolaryngology Research Center, Shiraz University of Medical Sciences, Shiraz, Iran

**Keywords:** salivary gland tumors, mismatch repair proteins, tumor-infiltrating lymphocytes, CD45RO ^+^ memory cells

## Abstract

**Introduction:**

Salivary gland tumors (SGTs) are uncommon lesions that account for 3 to 6% of head and neck cancers.

**Objective:**

To investigate mismatch repair (MMR) proteins, tumor-infiltrating lymphocytes (TILs), and CD45RO expression in salivary gland tumors (SGTs).

**Methods:**

Proteins MLH1, MSH2, MSH6, and PMS2 of the MMR proteins family and CD45RO were evaluated using immunohistochemistry (IHC). Hematoxylin and eosin-stained sections were scored to explore the rate of TILs.

**Results:**

None of the malignant and benign SGTs had partial or complete loss of at least one of the MMR proteins. Mucoepidermoid carcinomas, adenoid cystic carcinomas, salivary ductal carcinomas, and acinic cell carcinomas as malignant tumor types, and pleomorphic adenoma and Warthin's tumors as the most common benign tumors showed considerable differences in terms of the infiltration of TILs and CD45RO. Malignant tumors exhibited a notable difference in the infiltration of CD45RO ^+^ cells compared to benign ones. Both the tumor, node, metastasis (TNM) stage and the histological grade were shown to be linked with the infiltration status of CD45RO ^+^ cells.

**Conclusion:**

Our results show that MMR deficiency might be insignificant and less relevant in SGTs. However, differences in TIL rate and CD45RO expression indicate that each of the SGT tumor types may have distinguished immune microenvironments. Malignant SGTs have higher infiltration of activated immune cells, and, thereby, these cells can be considered as good indicators of patient's status.

## Introduction

Salivary gland tumors (SGTs) encompass a wide range of neoplasms that exhibit different morphological and clinical characteristics and are categorized as a type of head and neck cancer (HNC), accounting for 3 to 6% of all HNCs. 1 2

The rate of tumorigenesis in SGTs is low and located in the major and minor salivary glands. Major salivary glands consist of the parotid, submandibular, and sublingual glands. In addition, minor salivary glands are in the upper aerodigestive tract. A total of 80 to 85% of all SGTs occur in the parotid glands, over 10% originate from the submandibular glands, and the rest of them start in the sublingual or minor glands. 3 The majority of SGTs are benign and do not exhibit invasive behavior towards neighboring tissues. However, it is important to note that some of these tumors have the potential to recur and/or undergo malignant transformation and develop into cancerous growths. 4 The surgical removal of benign SGTs is the treatment of choice. Also, in some cases, radiotherapy will be done as an adjuvant therapy to inhibit tumor growth or malignant transformation. However, cases with malignant transformation are more dangerous and harder to treat. 4 5 Malignant SGTs are rare and estimated to be 5 to 7-fold less frequent than benign ones. 6 Malignant SGTs are distinguished by high locoregional failure, distant metastasis, and frequent genetic alterations. 7 In addition to surgical removal of tumors, chemotherapies, targeted therapies, and immunotherapy may play important roles in the clinical management of cases and improve the disease outcome. 8

In order to address the challenges posed by advanced and metastatic cancer, a number of novel immunotherapeutic interventions, especially checkpoint inhibitors, have been devised and put into practice. 9 For example, anti-PD-1/PD-L1 therapy has been approved for hot tumors with mismatch repair (MMR)-deficiency and high PD-1/PD-L1 expression. 10 Low-to-high expression of the immune checkpoints, including PD-1, PDLs, HLA-G, and LAG3, has been reported on tumor cells and/or tumor-infiltrating lymphocytes (TILs) of SGTs. 11 Our previous study indicated the simultaneous expression of PD-1 and PD-L1 in peripheral and central immune cells and tumor cells in both benign and malignant SGTs. 12 However, immunohistochemical expression of PD-L1 often fails to effectively predict the response to treatment, but, on the contrary, impairment in the MMR system and mutations and microsatellite instability (MSI) could be used as a proper tool to determine immunotherapy outcomes in different types of cancers, more especially in HNSCCs. 13

Mismatch repair system is a cellular process that guarantees the genomic stability of cells. 14 The MMR system is responsible for correcting spontaneous base-based mispairing and small insertion-deletion loops that arise during DNA replication or recombination. Deficiency of the MMR system is correlated with increased point mutations and MSI. Genomic instability caused by MMR deficiency is an important step in tumorigenesis. 15 MLH1, MSH2, MSH6, and PMS2 proteins are major components of the MMR system, and deficiency in any of these proteins can eliminate MMR activity. 16 Based on the current knowledge, MMR proteins seem to play an important role in cancer recurrence, precancerous lesions, and their progression to HNSCCs, but there are limited research works on their significance to SGTs. 17

Tumor progression is profoundly influenced by cancer cells' interactions with their surrounding environment, which determines the fate of a primary tumor. 18 Tumor-infiltrating lymphocytes reflect the host antitumor immune response. 19 CD45RO ^+^ memory TILs have garnered considerable interest due to their potential as a helpful biomarker for the prognosis of many cancer types, such as colorectal, gastric, esophageal, and hepatocellular carcinoma. 20 Based on the findings of our prior investigation, it has been demonstrated that CD45RO ^+^ TILs have the capacity to be integrated into clinicopathological prognostic indicators for individuals diagnosed with laryngeal squamous cell carcinoma. 21

In the present study, we analyzed a number of malignant and benign tumors to investigate the significance of MMR deficiency, CD45RO expression, and TIL infiltration in SGT patients and their association with the clinicopathological parameters of the disease.

## Material and Methods

### Study Population

The study population consisted of patients with SGTs who underwent surgical removal of their primary tumors as the initial treatment at Shiraz Ghadir Mother and Child Hospital, which is affiliated with Shiraz University of Medical Sciences in Shiraz, Iran. The patients were selected among those who had surgeries performed between 2014 and 2021. The hematoxylin and eosin (H&E) slides of patients were examined, and a proficient pathologist validated the histological diagnoses. The study included participants who possessed appropriate formalin-fixed paraffin-embedded (FFPE) tissue blocks and had accessible clinicopathological information. This study's procedure was authorized by Shiraz University of Medical Sciences' Ethics Committee (IR.SUMS.REC.1400.869).

### Immunohistochemistry (IHC)

Formalin-fixed paraffin-embedded blocks of tumor tissues were cut into 3-μm tissue sections by microtome and fixed on positively charged IHC slides. A total of five consecutive sections were obtained from each patient and subsequently subjected to deparaffinization. The sections were then heated in an oven set at a temperature of 61°C for 15 minutes, after which they were promptly immersed in fresh xylene for 30 minutes. The process of rehydration involves submerging the specimen in a series of decreasing concentrations of graded ethanol for 45 seconds. Heat-Induced Epitope Retrieval (HIRE) was employed for antigen retrieval, using Tris/EDTA pH 9.0 as the retrieval solution. To inhibit endogenous peroxidase activity, a solution of 3% hydrogen peroxide (H _2_ O _2_ ) was employed, whereas the application of 10% goat serum served to mitigate nonspecific binding. Anti-CD45RO (mouse monoclonal antibody, clone UCHL, 1/300 Dako, Denmark), anti-MLH1 (mouse monoclonal antibody, clone BS29, Master Diagnóstica, Granada, Spain), anti-PMS2 (mouse monoclonal antibody, clone EP51, Master Diagnóstica), anti-MSH2 (mouse monoclonal antibody, clone FE11, Master Diagnóstica), and anti-MSH6 (mouse monoclonal antibody, clone EP49, Master Diagnóstica) primary antibodies were added and incubated for 45 min in a humid chamber. The Master Polymer plus Detection System (Master Diagnóstica) was employed to facilitate the visualization process. This system resulted in the formation of a brown precipitate at the site of the antigen. The sections were subsequently counterstained with hematoxylin, dehydrated using a series of graded ethanol solutions, and permanently mounted using a mounting media (Entellan – Merck KGaA, Darmstadt, Germany).

### IHC Interpretation

An expert pathologist reviewed all tissue slides stained with H&E and IHC through direct microscopic observation and was blinded to the patients' clinicopathological information. The primary MMR proteins, namely MLH1, MSH2, MSH6, and PMS2, were evaluated using IHC. The comprehensive loss was determined to be the whole absence of discernible protein expression. The determination of partial loss was made by assessing the presence of patchy or incomplete staining, characterized by an intensity that was comparatively weaker than that of internal control. The presence of these proteins is indicated by the distinct nuclear staining. Patients with absent or partial expressions are classified as MMR deficient, while those with normal expression are classified as MMR proficient. Tumor-infiltrating lymphocytes and CD45RO ^+^ cell percentages were evaluated under a microscope via eye measurement. The H&E slide of each FFPE block scanned with an optical microscope and by morphological evaluation extent of TILs was scored as 1 (< 30%), 2 (30–60%), and 3 (> 60%) based on the proportion of lymphocyte areas to the total carcinoma area. The extent of CD45RO staining was scored as 1 (< 30%), 2 (30–60%), and 3 (> 60%) based on the proportion of positive staining areas to the total carcinoma area. The evaluation did not include necrotic areas, artifacts, and perivascular areas. The 60% cut-off point was used to divide TILs and CD45RO ^+^ cells into categories with low and high infiltration. 22

### Statistical Analysis

The IBM SPSS Statistics for Windows (IBM Corp., Armonk, NY, USA) version 20.0 was used for statistical analysis, and graphs were generated by GraphPad Prism 8.2.1 (GraphPad Software, Inc., San Diego, CA, USA). Differences in TILs and CD45RO ^+^ cell infiltration between different types of SGTs were studied with the Mann-Whitney U-test. All clinicopathological aspects of the study population were classified as binary variables, and differences in CD45RO ^+^ cells infiltration between them were investigated using the Chi-squared test and Fisher's exact test. The cut-off threshold of 60% was used to classify TILs and CD45RO ^+^ cells into low and high groups, respectively. The study employed the Spearman correlation test to examine the relationship between TILs and the expression of CD45RO. Significance was attributed to *p* -values that were less than 0.05.

## Results

### Patients

The study encompassed a total of 58 patients who had received a histological diagnosis of primary SGTs. Within this cohort of patients, a total of 28 benign tumors were seen, consisting of 15 cases of pleomorphic adenoma (PA) and 13 cases of Warthin's tumor (WT). Meanwhile, the remaining 30 individuals were diagnosed with malignant tumors. The sample of malignant tumors consisted of 9 cases of mucoepidermoid carcinoma (MEC), 7 cases of adenoid cystic carcinoma (AdCC), 5 cases of salivary ductal carcinoma (SDC), and 9 cases of acinic cell carcinoma (ACC). The primary site of the tumor was identified as the parotid gland in 53 cases, whereas in the remaining patients it was located in the submandibular gland. Their ages ranged from 18 to 81 years old, with a median of 49.5 years old at the time of surgery. There were 32 men and 26 women among the cases. The 8th edition of the TNM staging version for SGTs published by the American Joint Committee on Cancer (AJCC) in 2017 was used to determine histological tumor grade. Most of the patients had TNM stage III (n =12). Patient and tumor characteristics are reported in Table 1 .

**Table 1:** Clinicopathologic characteristics of patients with benign and malignant salivary gland tumors

	Total N (%)	Malignant N (%)	Benign N (%)
Age (years)			
≤ 50	29 (50)	16 (53.3)	13 (46.4)
> 50	29 (50)	14 (46.7)	15 (53.6)
Gender			
Female	26 (44.8)	16 (53.3)	10 (35.7)
Male	32 (55.2)	14 (46.7)	18 (64.3)
Primary tumor site			
Parotid	53 (91.4)	27 (90)	26 (92.9)
Submandibular gland	5 (8.6)	3 (10)	2 (7.1)
Tumor histology			
Pleomorphic adenoma	15 (25.9)		15 (53.6)
Warthin's tumor	13 (22.4)		13 (46.4)
Acinic cell carcinoma	9 (15.5)	9 (30)	
Adenoid cystic carcinoma	7 (12.1)	7 (23.3)	
Mucoepidermoid carcinoma	9 (15.5)	9 (30)	
Salivary duct carcinoma	5 (8.6)	5 (16.7)	
TNM classification			
I		3 (5.2)	
II		11 (19)	
III		12 (20.7)	
IV		4 (6.9)	
Tumor size			
≤ 3 cm	35 (60.3)	24 (80)	11 (39.3)
> 3cm	23 (39.7)	6 (20)	17 (60.7)
Grade			
Low		17 (56.7)	
High		13 (43.3)	
Lymph node invasion			
Yes	9 (16.1)	9 (30)	0 (0)
No	46 (83.9)	21 (70)	28 (100)
Perineural invasion			
Absent		11 (36.7)	
Present		17 (56.7)	
Unknown		2 (6.7)	
Lymph vascular invasion			
Absent		18 (60)	
Present		10 (33.3)	
Unknown		2 (6.7)	
Tumor extension			
Absent		11 (36.7)	
Present		18 (60)	
Unknown		1 (3.3)	

### Evaluation of the MMR Proteins Expression

**Fig. 1:**
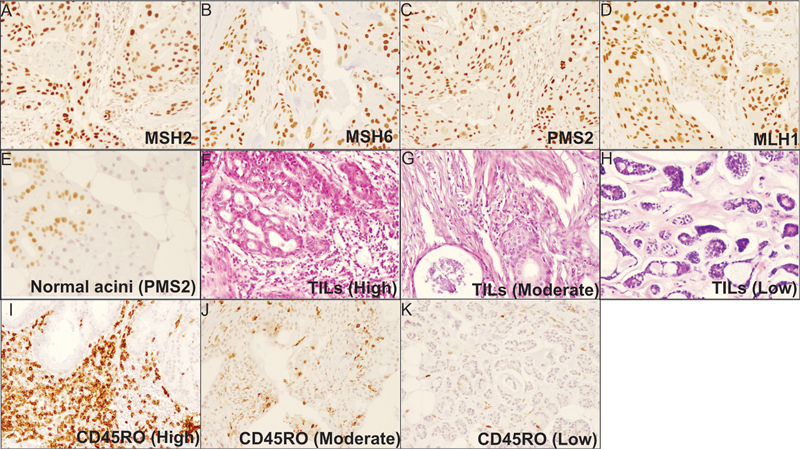
Expression pattern of MMR proteins and CD45RO+ TILS in SGT tissues using immunohistochemistry: A, B, C, and D represent MSH2, MSH6, PMS2, and MLH1 in mucoepidermoid carcinoma (MEC), with proficient MMR status. E represents PMS2 deficiency in normal Acinar cells from a patient with acinic cell carcinoma (ACC). F represents a high level of TILs in salivary duct carcinoma (SDC), G represents a moderate level of TILs in MEC, and H represents a low level of TILs in adenoid cystic carcinoma (AdCC). I represents a high level of CD45RO+ cells in SDC, J represents a moderate level of CD45RO+ cells in MEC, and K represents a low level of CD45RO+ cells in AdCC.

Expression of MMR proteins in SGTs was assessed with the IHC method. Among all malignant and benign tumors, none of them had partial or complete loss of at least one MMR proteins, including MLH1, MSH2, MSH6, and PMS2. One interesting thing about MMR status is that in all our samples which contained normal tissue (n = 46), normal acini were negative for PMS2 expression. Fig. 1 represents the expression pattern of studied markers.

### Evaluation of the TILs and CD45RO-Positive Cells Infiltration

First, we analyzed the rate of immune cells infiltration in different tumor types. Among benign tumors, patients with PA had significantly lower infiltration of TILs than cases with WT ( *p* < 0.0001, r = 0.981). Among malignant tumors, cases with MEC had significantly higher infiltration of TILs than cases with AdCC ( *p* = 0.015, r = 0.606).

**Fig. 2:**
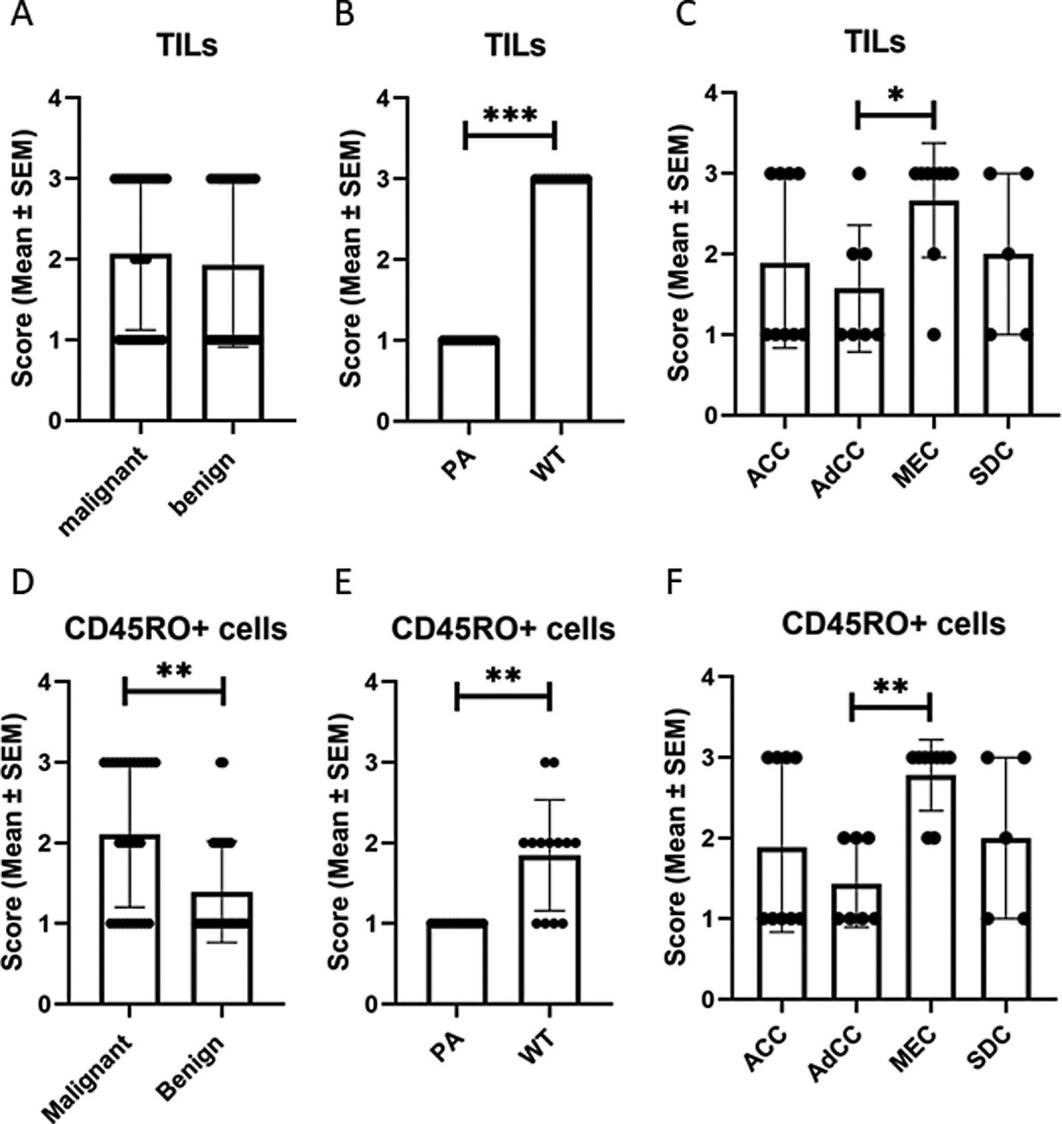
Comparison of TILs and CD45RO ^+^ cells infiltration in salivary gland tumors: There were no significant differences between benign and malignant SGTs ( **A** ). Patients with pleomorphic adenoma (PA) had significantly lower infiltration of TILs than cases with Warthin tumor ( *p* < 0.0001, r = 0.981) ( **B** ). Cases with MEC had significantly higher infiltration of TILs than cases with AdCC ( *p* = 0.015, r = 0.606) ( **C** ). Benign tumors had a significant difference in CD45RO ^+^ cell infiltration with malignant tumors ( *p* = 0.004, r = 0.381) ( **D** ). WT had a higher infiltration of CD45RO ^+^ cells than PA ( *p* < 0.001, r = 0.716) ( **E** ). Patients with MEC had significantly higher CD45RO ^+^ cell infiltration than AdCC ( *p* = 0.001, r = 0.807) ( **F** ). **Notes:** The data is presented as mean ± standard error of the mean (SEM) values. *, **, and *** differences are significant at 0.05, 0.01, and 0.001 levels respectively (2-tailed).

Benign and malignant tumors had a significant difference in CD45RO ^+^ cells infiltration, and the rate of infiltration was higher in malignant tumors ( *p* = 0.004, r = 0.381). Among benign tumors, WTs had higher infiltration of CD45RO ^+^ cells than PA ( *p* < 0.001, r = 0.716). Among malignant tumors, MEC had significantly higher CD45RO ^+^ cells infiltration than AdCC ( *p* = 0.001, r = 0.807). Fig. 2 represents the infiltration rate of TILs and expression level of CD45RO in different SGT tumor types.

### Correlation Analysis of TILs and CD45RO Expression

The results of Spearman's correlation analysis conducted on the malignant and benign SGTs dataset revealed a significant positive correlation between the level of TILs in malignant tumors and the expression of CD45RO by immune cells ( *p* < 0.001, CC = 0.935). But this correlation did not reach statistical significance in the case of benign tumors ( *p* = 0.124, CC = 0.298). Warthin tumors (n = 13) had high infiltration of TILs, but our data showed that in almost all cases (except 2), these tumors have a lower expression rate of CD45RO, and, on average, 50% of infiltrated cells were not positive for CD45RO expression.

### Association between CD45RO-Positive Cells Infiltration and Clinicopathological Features of Patients

Patients with benign tumors had a statistically significant relation between TILs and sex in such a way that females had significantly lower infiltration than males ( *p* < 0.001, r = 0.278). Furthermore, a correlation between age and the presence of TILs was seen in patients with benign tumors. Specifically, it was found that younger individuals had a lower level of TILs infiltration ( *p* < 0.001, r = 48). There was a significant association between the high infiltration of CD45RO ^+^ cells and the lower stages (TNM I and II) in malignant cases ( *p* = 0.03, R = 0.185). A similar pattern was observed in terms of TILs, although the observed differences have not reached statistical significance ( *p* = 0.07, r = 0.253). Higher infiltration of CD45RO ^+^ cells was also associated with lower tumor size and low-grade tumors ( *p* = 0.01, R = 0.147; *p* = 0.050, r = 0.21 respectively). Tables 2 3 present the association between TILs and CD45RO ^+^ cell infiltration with the clinicopathological features of patients.

**Table 2:** Comparison of clinicopathological characteristics based on CD45RO and TILs infiltration in benign salivary gland tumors

	Cases	TILs		*p*	CD45RO		*p*
		Low	High	(Odds ratio)	Low	High	(Odds ratio)
Age (years): n (%)							
≤ 50	13 (46.4)	12 (80%)	1 (7.7%)	*< 0.001*	13 (50%)	0 (0%)	0.484
> 50	15 (53.6)	3 (20%)	12 (92.3%)	(48)	13 (50%)	2 (100%)	(1.154)
Gender: n (%)							
Male	18 (64.3)	5 (33.3%)	13 (100%)	*< 0.001*	16 (61.5%)	2 (100%)	0.405
Female	10 (35.7)	10 (66.7%)	0 (0%)	(0.278)	10 (38.5)	0 (0%)	(0.889)
Tumor size: n (%)							
≤ 3 cm	11 (39.3%)	8 (53.3%)	3 (23.1%)	*0.102*	10 (38.5%)	1 (50%)	1.0
> 3 cm	17 (60.7%)	7 (46.7%)	10 (76.9%)	*(3.81)*	16 (61.5%)	1 (50%)	(0.625)

Abbreviation: TILs, tumor-infiltrating lymphocytes.

**Table 3:** Comparison of clinicopathological characteristics based on CD45RO and TILs infiltration in malignant salivary gland tumors

	Cases	TILs		*p*	CD45RO		*p*
		Low	High	(Odds ratio)	Low	High	(Odds ratio)
Age (years): n (%)							
≤ 50	16 (53.3)	9 (56.2%)	7 (50%)	0.732	9 (52.9%)	7 (53.8%)	0.961
> 50	14 (46.7)	7 (43.8%)	7 (50%)	(1.286)	8 (47.1%)	6 (46.2%)	(0.964)
Gender: n (%)							
Male	14 (46.7)	7 (43.8%)	7 (50%)	0.732	8 (47.1%)	6 (46.2%)	0.405
Female	16 (53.3)	9 (56.2%)	7 (50%)	(0.778)	9 (52.9%)	7 (53.8%)	(0.889)
Tumor size: n (%)							
≤ 3 cm	23 (76.7%)	11 (68.8%)	12 (85.7%)	*0.256*	11 (64.7%)	13 (100%)	*0.024*
> 3 cm	7 (23.3%)	5 (31.2%)	2 (14.3%)	*(0.367)*	6 (35.3%)	0 (0%)	(0.458)
Grade: n (%)							
Low	17 (56.7%)	7 (43.8%)	10 (71.4%)	*0.127*	7 (41.2%)	10 (76.9%)	0.050
High	13 (43.3%)	9 (56.2%)	4 (28.6%)	*0.311*	10 (58.8%)	3 (23.1%)	(0.21)
Lymph node invasion: n (%)							
No	21 (70%)	12 (75%)	9 (64.3%)	0.404	12 (70.6%)	9 (69.2%)	1.0
Yes	9 (30%)	4 (25%)	5 (35.7%)	(1.667)	5 (29.4%)	4 (30.8%)	1.067
TNM stage: n (%)							
Stages I and II	14 (46.7%)	5 (31.2%)	9 (64.3%)	0.07	5 (29.4%)	9 (69.2%)	*0.03*
Stages III and IV	16 (53.3)	11 (68.8%)	5 (35.7%)	(0.253)	12 (70.6%)	4 (30.8%)	0.185
Lymph vascular invasion: n (%)							
No	18 (64.3)	9 (60%)	9 (69.2%)	*0.705*	10 (62.5%)	8 (66.7%)	*1.0*
Yes	10 (35.7%)	6 (40%)	4 (30.8%)	(0.667)	6 (37.5%)	4 (33.3%)	(0.833)
Perineural invasion: n (%)							
No	17 (60.7%)	9 (60%)	8 (61.5%)	0.934	9 (56.2%)	8 (66.7%)	0.705
Yes	11 (39.3%)	6 (40%)	5 (38.5%)	(938)	7 (43.8%)	4 (33.3%)	(0.643)
Tumor extension: n (%)							
No	11 (37.9%)	5 (33.3%)	6 (42.9%)	0.597	5 (31.2%)	6 (46.2%)	0.466
Yes	18 (62.1%)	10 (66.7%)	8 (57.1%)	0.667	11 (68.8%)	7 (53.8%)	(0.530)

Abbreviations: TILs, tumor-infiltrating lymphocytes; TNM, Tumor, node, metastasis.

## Discussion

We investigated the expression profile of MMR proteins and CD45RO ^+^ cells infiltration in a cohort of 58 benign and malignant SGTs. Deficiency in the MMR system leads to MSI, which can induce frameshift mutations. When these mutations occur within coding sequences, they can give rise to tumor-specific “neoantigens”—abnormal proteins that are identifiable by the immune system as foreign entities. Also, MSI indicates a unique carcinogenic mechanism characterized by changes in the expression of tumor suppressor genes and the activation of oncogenes, ultimately resulting in the malignant transformation. 17 In our study, none of the patients had MMR deficiency, and it seems that it may be an insignificant and less relevant marker for the characterization of these patients. However, in all of our samples which contained normal tissue, normal acini were negative for PMS2 expression. The identification of negative PMS2 expressions within normal acini is a noteworthy discovery since it suggests a potential heightened susceptibility of acinar cells to carcinogenesis. However, it should be noted that tumorigenesis in acinar cells can cause ACC, and in our cohort with 9 cases of ACC, none of them was MMR deficient. Further investigation is warranted to gain a comprehensive understanding of the underlying processes via which negative PMS2 influences the pathophysiology of acinar cells, particularly considering that none of the tumor samples analyzed in our study exhibited a deficiency in PMS2 expression.

We assessed the infiltration profile of TILs and CD45RO ^+^ cells in SGTs. These could be considered as an indicator of immune response in solid tumors. 23 We found that malignant tumors had higher infiltration of CD45RO ^+^ cells than benign tumors. Also, our results showed a positive correlation between TILs in malignant tumors and immune cell CD45RO expression. As we know, the immunogenicity of a tumor is the ability to induce adaptive immune responses. 24 CD45RO is typically observed on activated and memory T cells, which serve as an indicator of an activated and mature immune response against tumor cells. 25 These observations likely suggest that malignant tumors may exhibit a higher degree of immunogenicity compared to benign tumors, which leads to higher activation of TILs in malignant tumors. Warthin tumor and PA are both classified as benign tumors; nevertheless, they exhibit distinct immunological microenvironments. For example, the study reveals that WT exhibits a very immune-rich milieu, whereas PA tumors exhibit limited infiltration of immune cells into the microenvironment. This finding may be because of the nature of WT, which consists of oncocytic epithelium and lymphoid stroma. 26 This implies that the recruitment of immune cells in the microenvironment of WT may not be affected by the presence of tumor, but under normal conditions, these immune cells are able to be recruited to lymphoid tissues. In the category of malignant tumors, instances of MECs exhibited a notably greater degree of infiltration by TILs and CD45RO in comparison to cases of AdCC. Adenoid cystic carcinoma is considered to be a poorly immunogenic tumor for a number of reasons, including low levels of MHC expression, immunosuppressive, and hostile microenvironment to immune cells. 27 28

Furthermore, our results indicated an association between TILs and gender and age among patients diagnosed with benign tumors. Specifically, males and old patients exhibited considerably higher levels of TIL infiltration compared to females and young patients, respectively. However, significant differences were not seen in terms of CD45RO ^+^ cell infiltration. Studies have shown that women tend to have higher levels of TILs than men. This may be due to the fact that women have a stronger immune response than men. 29 Also, based on immunosenescence, a phenomenon that describes the alterations in older people's immune systems, aged patients tend to have lower levels of TILs. 30 Our result does not support these phenomena. As mentioned above, the presence of TILs in benign tumors does not serve as a reliable indicator for assessing the extent of immune response. The findings may support the fact that the presence of these cells can be attributed to the native characteristics of the tissue in which the tumor originated rather than only to the inflammation induced by the tumor. In the context of SGT benign cases, it is advisable to employ CD45RO ^+^ cells as the foundation for examining the immune system's reaction.

Among the therapeutically significant aspects of cancer patients, histological grade is one of the most critical elements. Pereira et al. reported higher infiltration of CD8 ^+^ TILs in low-grade, oral squamous cell carcinoma than cases with high-grade tumors. 31 Wang et al. reported the same association of CD4 and CD8 ^+^ TILs with advanced hypopharyngeal squamous cell carcinoma. 32 It seems that an active immune response can potentially contribute to the prevention of cancer development and growth.

In our results, the lower number of CD45RO ^+^ TILs was associated with aggressive phenotypes of the disease, including advanced TNM-stage and increased tumor size. Tumor growth is not an autonomous cell process and is influenced by its surrounding microenvironment. 33 Immune cells in the TME can affect the growth and evolution of cancerous cells. CD45RO ^+^ memory T-cells have a significant impact on the immune response against tumors. These cells possess the capability to identify and eliminate malignant cells associated with cancer. Memory T-cells can be activated in low levels of antigen presentation as well as costimulatory agents. These cells can survive for long terms. 20 Based on these observations, it may be inferred that the immune response inside the TME exerts a regulatory influence on tumor development and progression, as well as on the acquisition of aggressive phenotypes, despite its failure to eliminate clinically evident malignancies. So, CD45RO ^+^ cells can be indicators of host anti-tumor immune status, and a higher number of them may control tumors or delay the acquiring of aggressive phenotypes. 21

## Conclusion

Our results showed that MMR deficiency might be insignificant and less relevant in SGTs. PMS2 deficiency in normal acinar cells needs to be evaluated, and its effect on possible tumorigenesis of these cells needs to be determined in future studies. Differences in the TILs rate and CD45RO expression indicate that each of the SGT tumor types may have distinguished immune microenvironments. Malignant SGTs have higher infiltration of activated immune cells, and these cells can be considered good indicators of the patient's status.
